# Prevalence of Chronic Kidney Disease as a Marker of Hypertension Target Organ Damage in Africa: A Systematic Review and Meta-Analysis

**DOI:** 10.1155/2021/7243523

**Published:** 2021-10-11

**Authors:** Samuel O. Ajayi, Udeme E. Ekrikpo, Anyiekere M. Ekanem, Yemi R. Raji, Okechukwu S. Ogah, Dike B. Ojji, Ugochi S. Okpechi-Samuel, Kwazi C. Z. Ndlovu, Aminu K. Bello, Ikechi G. Okpechi

**Affiliations:** ^1^Department of Medicine, College of Medicine, University of Ibadan, Ibadan, Oyo State, Nigeria; ^2^Division of Nephrology and Hypertension, University of Cape Town, Cape Town, South Africa; ^3^Renal Unit, Department of Internal Medicine, University of Uyo, Uyo, Nigeria; ^4^Department of Community Health, Faculty of Clinical Sciences, University of Uyo, Uyo, Nigeria; ^5^Department of Medicine, Faculty of Clinical Sciences, University of Abuja, and University of Abuja Teaching Hospital, Gwagwalada, Nigeria; ^6^Department of Internal Medicine, Federal Medical Centre, Jabi, Abuja, Nigeria; ^7^Kidney and Hypertension Research Unit, University of Cape Town, Cape Town, South Africa; ^8^Division of Nephrology and Immunology, Faculty of Medicine and Dentistry, University of Alberta, Edmonton, Alberta, Canada

## Abstract

**Introduction:**

Hypertension is a major global cause of cardiovascular disease and death with rising worldwide prevalence, particularly in low-income countries. With low awareness, poor treatment, and low control of hypertension in Africans, there is an increased number of patients with target organ damage (TOD), especially chronic kidney disease (CKD), as a consequence of hypertension. The aim of our study is to assess the prevalence of CKD from studies in Africa reporting TOD related to hypertension.

**Methods:**

We performed a search of PubMed/MEDLINE, Web of Science, EBSCOhost, and African Journals Online (AJOL) for studies reporting on CKD as TOD in patients with hypertension. The pooled estimate of CKD was then presented by subregions, age group, eGFR equations, and urban or rural location.

**Results:**

We identified 1,334 articles from which 12 studies were included for quantitative analysis. The studies included 5297 participants from 6 countries (Ghana, Nigeria, Uganda, Tanzania, Democratic Republic of Congo, and South Africa). The pooled prevalence of CKD was 17.8% (95% CI 13.0–23.3%), and CKD was significantly more prevalent in West Africa (21.3% (95% CI: 16.1–27.0); *p* < 0.0001) and in studies conducted in urban settings (*p* < 0.001). CKD prevalence was not significantly different by type of GFR equation or age.

**Conclusion:**

This study reports a high prevalence of CKD related to hypertension with a higher prevalence in urban than rural areas. This emphasizes the role of hypertension in causing kidney damage, and the need for strategies to improve awareness, treatment, and control of hypertension in Africans. This study is registered with PROSPERO registration number CRD42018089263.

## 1. Introduction

Hypertension is a prevalent worldwide public health problem and an important modifiable risk factor for cardiovascular disease (CVD) and chronic kidney disease (CKD). The prevalence of hypertension in the global adult population was estimated to be 31.1% (95% CI: 30.0%–32.2%) in 2010, representing 1.38 billion people who were affected worldwide [1, 2]. In Africa, this amounted to a prevalence of 36.9% (33.2–40.5%) in men and 36.3% (32.9–39.6%) in women, representing 64.8 million men and 63.8 million women, respectively [2]. Despite the extensive availability of effective treatment options, blood pressure (BP) control remains suboptimal [3], especially in low-and middle-income countries with significant consequences on morbidity and mortality. Between 1990 and 2013, the global prevalence of CKD was noted to have increased by 48%, from 318.7 million to 471.9 million (about 2.1% per year) [3]. Within this same period, there was a 26.8% increase in number of CKD cases attributed to hypertension. The prevalence of CKD in sub-Saharan Africa (SSA) has been reported to be 13·9% (95% CI 12·2–15·7); this, however, included CKD from all known risk factors (human immunodeficiency virus (HIV), diabetes mellitus, and hypertension) [4]. The United States Renal Data System (USRDS) has consistently shown that CKD or kidney failure due to hypertension is more common in African Americans than in Hispanics or Caucasians [5].

There are subtle differences in the pathogenesis of hypertension between populations of African ancestry and Europeans, and such differences may account for the varied course of hypertension in these populations [6]. For instance, evidence suggests a more aggressive course of hypertension in causing early target organ damage (TOD) in black populations compared to Caucasians [7, 8]. The prevalence of hypertensive TOD in African Americans has been shown to be significantly higher than in non-Hispanic whites (41% vs 28%), more common at an earlier age and more difficult to control in blacks than in whites [9, 10]. Several factors may be responsible for the excess TOD associated with hypertension amongst people of African ancestry, including but not limited to differences in salt sensitivity (associated with increased water retention and sodium excretion) [11, 12], genetic factors like apolipoprotein A1 (APOL1) polymorphisms [13, 14], and circulating cytokines, e.g., transforming growth factor beta (TGF-*β*) [15] and socioeconomic factors [16].

Given that blood pressure (BP) is a modifiable risk factor for CKD, it is important to identify the contribution of hypertension to CKD in Africans, especially as access to treatment for CKD and kidney failure is severely limited [17] with extremely poor outcomes in Africa [18]. This study aims to comprehensively and systematically assess the prevalence of CKD from studies that attributed kidney disease to hypertension as the primary cause.

## 2. Methods

The Preferred Reporting Items for Systematic Review and Meta-Analysis [19, 20] was used to present this study. This study was registered with the International Prospective Register of Systematic Reviews, PROSPERO, with registration number CRD42018089263. Ethics approval was not required for this study.

### 2.1. Eligibility Criteria

**Figure 1:**
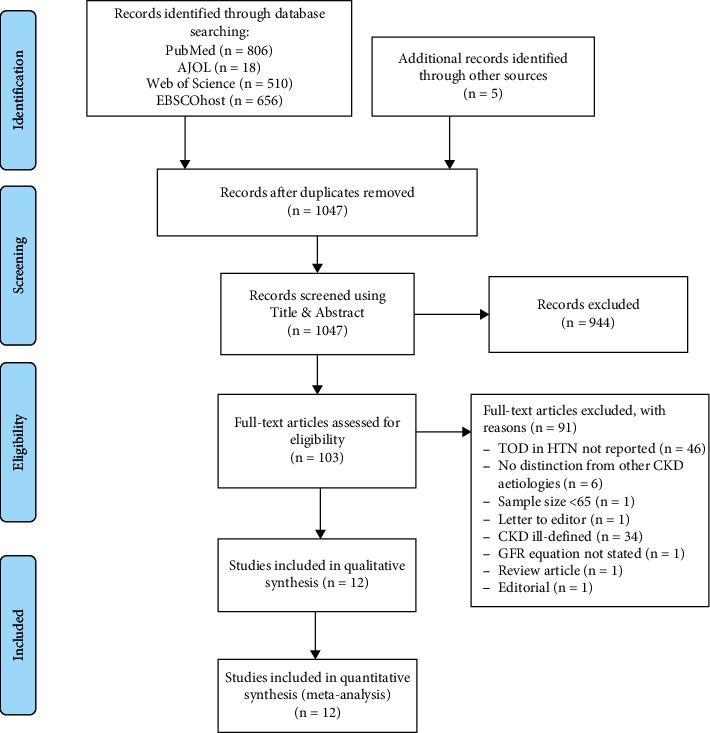
PRISMA flow diagram for study selection.

Published literature between January 1995 to December 2019 was included. We selected 1995 as the start year for our search as the definitions and stages of CKD were developed subsequently. We included all studies (without language restrictions) that reported CKD (based on clinical assessment) as TOD of hypertension in adults 18 years or older and included studies conducted in Africa. Hypertension was defined as systolic BP (SBP) ≥140 mmHg and/or diastolic BP (DBP) ≥90 mmHg [21]. We reported the prevalence of CKD (estimated GFR ≤60 mL/min/1.73 m^2^) as documented by the authors using any of the standard creatinine-based formulae to estimate the glomerular filtration rate (GFR): Chronic Kidney Disease Epidemiology (CKDEPI) [22], Modification of Diet in Renal Disease (MDRD) [23], and Cockcroft–Gault (CG) [24]. We excluded studies on children, special populations (e.g., HIV-positive patients only, studies on pregnant women, or studies in elderly alone), studies on hypertension in Africans living outside Africa, studies not reporting the prevalence of TOD, and studies in which it was not possible to extrapolate the prevalence of kidney-related TOD even after contacting the authors for data.  Figure 1 shows the Preferred Reporting Items for Systematic Reviews and Meta-Analyses (PRISMA) flowchart summarizing the study selection process.

### 2.2. Search Strategy for Identifying Relevant Studies

We performed a search of PubMed/MEDLINE, Web of Science, EBSCOhost, and African Journals Online (AJOL). Medical subject headings (MESH), including “prevalence,” “hypertension,” blood pressure,” “hypertensive,” “hypertensive target organ damage,” “chronic kidney disease,” “kidney disease,” kidney damage,” kidney failure,” “target organ damage,” “Africa,” “sub-Saharan Africa,” ‘Africa South of Sahara,” and all African countries by names were used in the search and modified for each database, as necessary. We adapted the MEDLINE search strategy for other databases (Supplementary Table S1).

### 2.3. Study Selection

The titles and abstracts of identified studies were independently screened by SA and UE, and full texts were also screened by both reviewers. All conflicts were resolved by a third reviewer (IGO).

### 2.4. Data Extraction and Management

Data extraction was done independently and in duplicates by SA and UE, and disagreement was resolved by consensus by SA and UE, and when necessary, in consultation with IGO. The following data variables were extracted from selected studies: first author's name, year of publication, country and subregion of study, race of study participants, study design, total sample size, sample size of study participants with hypertension, gender proportion, median age, mean body mass index (BMI), mean SBP, mean DBP, eGFR equation used for assessment, number, and proportion of participants with CKD.

### 2.5. Risk of Bias in Individual Studies

To assess the quality of methodology of the studies included for analysis, a modified 9-point rating system by Stanifer et al. [4] was used (Supplementary Table S2). Representativeness of the study participants, adequacy of sample size, and confounders of the relationship between hypertension and CKD were criteria for rating. Ratings with scores higher than 6, 4–6, and less than 4 were judged as high, medium, and low quality, respectively (Supplementary Table S3).

### 2.6. Statistical Analysis and Synthesis of Results

STATA 16 (Stata Corp., 2019. Stata Statistical Software: Release 16. College Station, TX: Stata Corp., LP) was used for statistical analysis.

The prevalence of TOD in hypertensives was performed using meta-analytic techniques. The study-specific estimates derived from the DerSimonian–Laird random-effects model [25] were pooled to estimate the prevalence of CKD according to the three eGFR equations: CKD-Modification of Diet in Renal Disease (MDRD), [23] CKD-Cockcroft–Gault (CG) [24], and CKD-Epidemiology Collaboration (CKDEPI) [23, 26]. To minimize the effect of extreme prevalence on the overall estimate, the Freeman–Tukey double arcsine transformation method was used to stabilize the individual study variances [27, 28]. Publication bias was assessed using funnel plots and Egger's test [29]. The *I*^2^ statistic was used to determine the heterogeneity between studies [30]. Subgroup analysis was performed using the *Q*-test based on ANOVA. Comparison of prevalence of hypertension and TOD between age groups, gender, location (urban versus rural), and region (West Africa versus East Africa versus Southern Africa) was done.

## 3. Results

### 3.1. General Characteristics of Included Studies

Our initial search identified 1,334 articles. After removal of duplicates, 1,045 articles were screened by titles and abstracts, from which 103 were selected for full-text review and 12 studies identified for inclusion. Of the eligible studies for inclusion [31–42], one study [40] reported prevalence of CKD using 3 different equations; hence, prevalence reported from each equation was recorded as a separate study (Table 1). Overall, the studies included 12,197 subjects, of which 5,297 (43.4%) were hypertensives in 6 sub-Saharan African countries. Seven of the studies were from West Africa [31, 34, 35, 39–42], 3 studies from East Africa, [32, 34, 37], with 1 study each from Central Africa [38] and Southern Africa [33], respectively. All the studies had a cross-sectional design and were all community-based assessments, and none of the studies included subjects of other race other than Black Africans. Eleven of the studies (91.7%) were of medium quality [31–34, 36–42], while one (8.3%) was of low quality [35] (Table 1). The mean age of the patients ranged from 31.0 years to 57.9 years, with the proportion of male subjects ranging from 21.7% to 45.7%. Five of the studies [31, 34, 37, 40, 42] had participants from both rural and urban communities, five studies were conducted in urban communities [33, 35, 38, 39, 41], while two studies were from predominantly rural dwellers [32, 36]. There was no evidence of publication bias (*p* value of the Egger test = 0.19 (Figure S1)).

### 3.2. Estimates of Hypertension, Systolic, and Diastolic Blood Pressures

The mean systolic blood pressure ranged from 135.9 mmHg [41] to 175.4 mmHg [39] (pooled SBP estimate of 152.0 (95% CI: 144.9–159.0 mmHg)), while the diastolic blood pressure ranged from 87.0 mmHg [41] to 101.2 mmHg [39] (pooled DBP estimate of 94.1 (95% CI: 90.2–97.9 mmHg)).

### 3.3. Prevalence of Kidney Disease from the Included Studies

**Figure 2:**
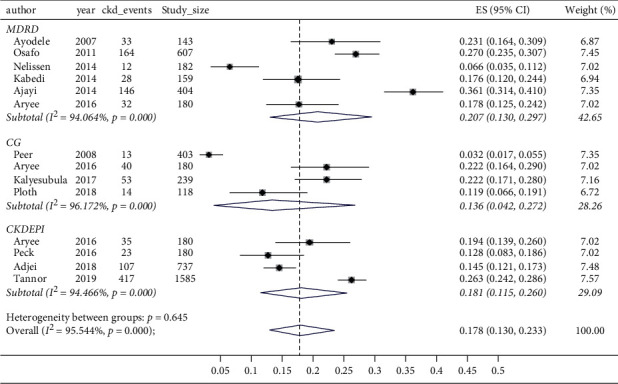
Forest plot and meta-analysis of estimates of CKD prevalence according to eGFR equations. MDRD, modification of diet in renal disease; CG, Cockcroft–Gault; CKDEPI, Chronic Kidney Disease Epidemiology Collaboration.

**Figure 3:**
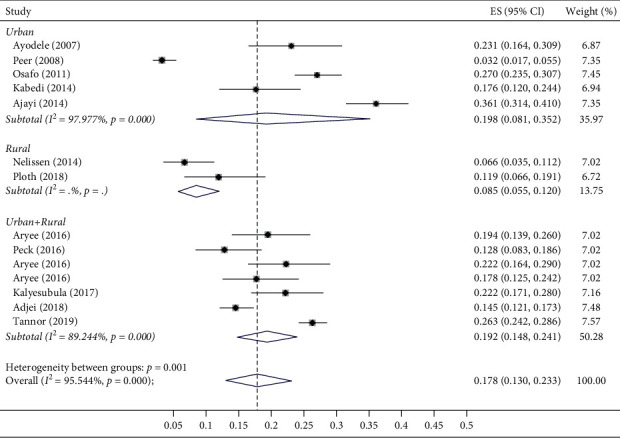
Forest plot showing the prevalence of CKD in urban, rural, and mixed populations. ES, effect size; CI, confidence interval.

Overall, the pooled prevalence of CKD among patients with hypertension in sub-Saharan Africa was 17.8% (95% CI 13.0–23.3%, *I*^2^ = 95.5%, *p* < 0.001) (Figure 2). Four studies reported the prevalence with the Cockcroft–Gault [32, 33, 37, 40], 6 studies with the MDRD equation [35, 36, 38–41], and 4 studies using the CKDEPI equation [31, 34, 40, 42]. The prevalence of CKD was noted to be different based on the equation used for reporting CKD: 13.6% (95% CI: 4.2–27.2%) using the Cockcroft–Gault equation, 18.1% (95% CI: 11.5–26.0%) using the CKDEPI equation, and 20.7% (95% CI: 13.0–29.7%) using the MDRD equation; however, these were not significantly different (*p*=0.65; Figure 2). The prevalence of CKD among hypertensives was also noted to be significantly higher for studies conducted in an urban setting (19.8% (95% CI: 8.1–35.2%)) than for those in rural settings (8.5% (95% CI: 5.5–12.0%); *p*=0.001) (Figure 3).

**Figure 4:**
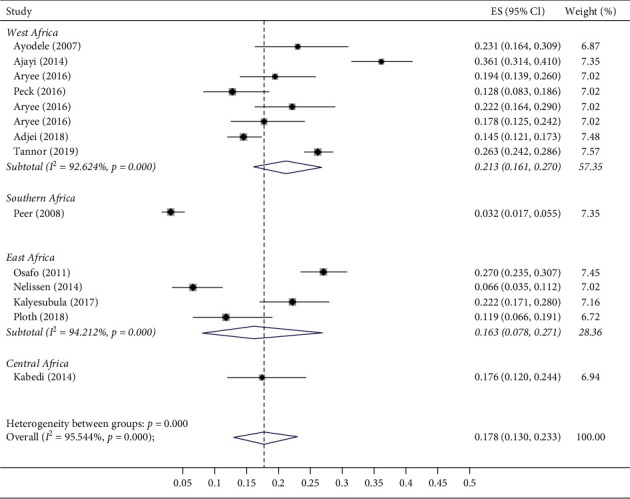
Forest plot and meta-analysis of prevalence of CKD in subregions of Africa. ES, effect size; CI, confidence interval.

When assessed according to region, studies from West Africa had the highest pooled prevalence of CKD (21.3% (16.1–27.0)), while the one study from Southern Africa had the lowest (3.2% (95% CI: 1.7–5.5%)), with a significant difference in prevalence across regions (*p* < 0.001, Figure 4). There was no significant difference in the pooled prevalence of CKD by age (*p*=0.76, Supplementary Figure 2) or by the gender (*p*=0.95, Supplementary Figure 3).

## 4. Discussion

Hypertension is a major global cause of morbidity and mortality and is associated with TOD, including CKD due to low awareness, poor treatment, and low control of high BP, especially in African countries [1, 2]. In this systematic review and meta-analysis of 12 African studies that included 5,297 participants from 6 countries, the pooled prevalence of CKD was found to be 17.8% (95% CI: 13.0–23.3%) with the highest prevalence seen in West Africa (21.3% (95% CI: 16.1–27.0)). Our study also showed the prevalence of hypertension-related CKD to be higher in urban than rural settings with no significant difference in prevalence from reporting with different CKD equations and age.

Several studies continue to show an upward projection and trend in the prevalence of hypertension across all world regions, particularly in developing regions such as Africa [43]. Given the relationship between BP and kidney damage, the impact of such rising trend on CKD prevalence and outcomes is likely to be devastating in Africa where availability and accessibility of resources to manage CKD is limited [44]. With an estimated 130 million adult Africans with hypertension [2], our observed prevalence of 17.8% for CKD due to hypertension is very high as there are other significant causes of CKD in Africans, including HIV, diabetes mellitus, nephrotoxins, and autoimmune disorders. Kaze et al. reported a prevalence of 4.6% (95% CI: 3.3–6.1) for CKD stages 3–5 in the general population of Africa and a prevalence of 9.1% (95% CI 6.6–11.9) in patients with HIV, 17.9% (95% CI 10.9–26.1) in patients with hypertension, and 22.0% (95% CI 16.1–28.6) in patients with diabetes mellitus [45]. Another study also found CKD related to hypertension to be as high as 24% in Africans [4]. As hypertension is one of the commonly modifiable risk factors for CKD, the high prevalence of CKD due to hypertension in our study is an important reason to increase advocacy for more noncommunicable disease (NCD) prevention programs as well as programs that specifically address hypertension awareness, treatment, and control in Africans. Data from the International Society of Hypertension May Measurement Month screening program for 2019 showed that of all world regions, Africa had the lowest proportion of hypertensives who were aware (42.7%), lowest proportion on treatment (34.5%), and lowest proportion of hypertensives with controlled BP (17%) [1]. The proportion of controlled hypertensives is much lower than the target of 25% set by the Pan African Society of Cardiology (PASCAR) on the roadmap for hypertension control by 2025 [46]. Although there is still time, such data highlight the magnitude of the problem of BP control and its consequences in Africa. What still needs to be seen are deployment of effective strategies aimed at reducing the burden of hypertension including awareness campaigns, identifying effective lifestyle measures, and ensuring availability of cost-effective medicines to improve BP control.

Our study also found that the West African subregion had the highest pooled prevalence of CKD (21.3% (16.1–27.0%); *p* < 0.0001). This is consistent with general population reports from previous studies showing higher CKD prevalence in West Africa [4, 39, 47]. The reason for this higher prevalence in West Africans may be related genetic factors. The high prevalence of CKD among African Americans has been attributed to the presence of the high-risk renal variant of APOL1 gene, and frequency of this gene has been shown to be high in Nigeria and Ghana [48]. The APOL1 gene has been shown to be independently associated with hypertension and CKD [49, 50]. We did not study specific races, and indeed, the works we included in our studies did not look at races in Africa. There are ongoing studies that are looking at the specific genetic aetiologies among Africans with hypertension and chronic kidney diseases. These include the H3Africa Kidney Research Network which is looking at the APOL1 polymorphisms in kidney disease. Differences in other factors that play a role in hypertension prevalence such as diet, salt intake, smoking, socioeconomic status, and availability of primary care services for healthcare could have contributed to the differences in regional CKD prevalence from our study [51, 52].

Furthermore, we also found a higher prevalence of CKD among the urban populations compared to rural populations. This is largely consistent with observations from studies in Africa of higher prevalence of hypertension in urban than rural settings [53]. Epidemiological transitions associated with increased urbanization have been shown to be directly associated with an increased prevalence of hypertension likely due to increased smoking, excessive alcohol consumption, increased consumption of processed food and salt, and a stressful lifestyle [54, 55]. For these reasons, studies in Africa have also shown better BP control in rural than urban settings. Despite this, it is important that NCD strategies are implemented to reduce the burden of hypertension in SSA.

Finally, our study identified nonsignificantly different CKD prevalence rates based on the different equations used for assessing CKD from the included studies. Given that the current equations in use were not developed in sub-Saharan African populations, uncertainties remain about the best equation for assessing GFR in Africans. The accuracy of current equations remains limited at an individual level, particularly due to differences in muscle mass. Other studies have also reported differences in CKD prevalence using these equations with an overestimation using the CG equation [27, 45]. This suggests the need to validate eGFR equations according to patient characteristics in Africa. It is hoped that ongoing prospective studies will be able to resolve this issue [56].

There were some limitations to our study. First, the absence of reporting serial measurements of serum creatinine and or GFR at least 3 months apart to define CKD is notable. Severe hypertension can be associated with acute kidney dysfunction; therefore, it may not be possible to distinguish such cases from CKD. Thus, the use of a single GFR estimation to assess CKD prevalence is likely to overestimate the prevalence [57]. This has been a limitation of many such studies reported from Africa and could be due to lack of resources for further evaluations [45]. However, it is highly unlikely in our study that the cases identified were acute kidney dysfunction related to uncontrolled hypertension as such cases are usually identified in a hospital and not in the community setting. Second, the inclusion of studies of low and moderate qualities accounted for significant heterogeneity which was not completely explained by subgroup analyses. Although this could be due to between-study differences in methodology and population structure, it may also represent the true country differences in disease burden. Finally, the primary studies included lacked data on important covariates that could be useful for further analysis and to help explain some of the differences observed in our study, including methods of ascertainment of hypertension-related CKD such as albuminuria and histological evidence. Most of the studies did not include albuminuria and at best only documented dipstick proteinuria which has a limited value. With regards to the regions, we identified only a few studies from South and Central Africa. Despite these limitations, an important strength of this study is the use of comprehensive and systematic methods in identifying and evaluating studies to be included in this review. Another strength of this study lies in identifying not just the prevalence of hypertension-attributable CKD but factors associated with this increase like urban lifestyle and being of West African origin. This ensures the validity of our findings which add important data to the literature on hypertension in Africans.

## 5. Conclusion

Our study shows that the prevalence of CKD is high amongst hypertensive patients in Africa, particularly for those in urban than in rural areas. Given that blood pressure is a modifiable risk factor for CKD, strategies to improve hypertension awareness, treatment, and control should be implemented in African countries. Such strategies should include strengthening NCD structures in each country and ensuring availability of cheap but effective drugs for hypertension treatment.

## Figures and Tables

**Table 1:** Clinical characteristics of included studies.

Author	Year of publication	Country	Region	Study design	Study location	Study setting	Female (%)	Total sample	No with HTN	No with CKD	Mean SBP (mmHg)	Mean DBP (mmHg)	eGFR formula	Method of CKD assessment	Quality
Ayodele et al. [39]	2007	Nigeria	West Africa	Cross-sectional	Urban	Community	55.9	143	143	33	175.36	100.01	MDRD	2x GFR (3 months)	Medium
Peer et al. [33]	2008	South Africa	Southern Africa	Cross-sectional	Urban	Community	55	403	403	13	146 ± 27	89 ± 13	CG	1x GFR	Medium
Osafo et al. [35]	2011	Ghana	West Africa	Cross-sectional	Urban	Community	78.25	607	607	164	150	90	MDRD	1x GFR + 1x UPCR	Low
Ajayi et al. [41]	2014	Nigeria	West Africa	Cross-sectional	Urban	Community	51.3	628	404	146	135.9 ± 27.4	87.0 ± 16.3	MDRD	1x GFR	Medium
Nelissen et al. [36]	2014	Nigeria	West Africa	Cross-sectional	Rural	Community	64.8	927	182	12	154.5 ± 21.2	96.3 ± 11.8	MDRD	1x GFR	Medium
Kabedi et al. [38]	2014	DRC	Central Africa	Cross-sectional	Urban	Community	54	159	159	28	159.1	95.1	MDRD	1x GFR	Medium
Aryee et al. [40]	2016	Ghana	West Africa	Case-control	Urban/rural	Community	58.89	241	180	40	154.69	101.24	CG	1x GFR	Medium
Aryee et al. [40]	2016	Ghana	West Africa	Cross-sectional	Urban/rural	Community	58.89	241	180	32	154.69	101.24	MDRD	1x GFR	Medium
Aryee et al. [40]	2016	Ghana	West Africa	Cross-sectional	Urban/rural	Community	58.89	241	180	35	154.69	101.24	CKDEPI	1x GFR	Medium
Peck et al. [34]	2016	Tanzania	East Africa	Cross-sectional	Urban/rural	Community	54.3	1095	180	23	NR	NR	CKDEPI	1x GFR	Medium
Kalyesubula et al. [37]	2017	Uganda	East Africa	Cross-sectional	Urban/rural	Community	66.53	955	239	53	NR	NR	CG	1x GFR + 1x dipstick	Medium
Adjei et al. [42]	2018	Ghana	West Africa	Cross-sectional	Urban/rural	Community	65.8	2524	737	107	NR	NR	CKDEPI	1x GFR + 1x UPCR	Medium
Ploth et al. [32]	2018	Tanzania	East Africa	Cross-sectional	Rural	Community	65.97	739	118	14	NR	NR	CG	1x GFR	Medium
Tannor et al. [31]	2019	Ghana	West Africa	Cross-sectional	Urban/rural	Community	76.25	3294	1585	417	140.92	NR	CKDEPI	1x GFR + 1x dipstick	Medium

SBP, systolic blood pressure; DBP, diastolic blood pressure; HTN, hypertension; eGFR, estimated glomerular filtration rate, CKP-EPI, Chronic Kidney Disease Epidemiology Collaboration; MDRD, modification of diet in renal disease; CG, Cockcroft–Gault; NR, not reported; 1x, performed once; 2x, performed twice.

## Data Availability

The data supporting this SYSTEMATIC REVIEW or META-ANALYSIS are from previously reported studies and datasets, which have been cited. The processed data are included within the article.

## Supplementary Materials

Supplementary Materials Supplementary Table S1: MEDLINE search strategy. Supplementary Table S2: scoring criteria for quality of studies (adapted from Stanifer et al., Lancet Glob Health 2014; 2 *e*174–181). Supplementary Table S3: risk of bias assessment. Supplementary Figure 1: funnel plot to assessing publication bias (ES, effect size). Supplementary Figure 2: prevalence of CKD categorized by age groups (ES, effect size; CI, confidence interval). Supplementary Figure 3: prevalence of CKD categorized by female gender preponderance (ES, effect size; CI, confidence interval).
