# Supplementary material for: Prevalence of Chronic Kidney Disease as a Marker of Hypertension Target Organ Damage in Africa: A Systematic Review and Meta-Analysis
Source: Int J Hypertens. 2021 Oct 11;2021:7243523. doi: 10.1155/2021/7243523 (PMC8523261; doi:10.1155/2021/7243523)
Supplement: Supplementary Materials — Supplementary Table S1: MEDLINE search strategy. Supplementary Table S2: scoring criteria for quality of studies (adapted from Stanifer et al., Lancet Glob Health 2014; 2 e174–181). Supplementary Table S3: risk of bias assessment. Supplementary Figure 1: funnel plot to assessing publication bias (ES, effect size). Supplementary Figure 2: prevalence of CKD categorized by age groups (ES, effect size; CI, confidence interval). Supplementary Figure 3: prevalence of CKD categorized by female gender preponderance (ES, effect size; CI, confidence interval). [file 7243523.f1.docx]

**Supplementary materials:**

**Supplementary Table S1:** MEDLINE search strategy

| Search ((Prevalence) OR Incidence) OR Frequency |
| --- |
| Search ((((Chronic kidney disease) OR Chronic kidney insufficiency) OR Chronic renal failure) OR chronic renal insufficiency) OR CKD |
| Search (((Hypertension) OR High blood pressure) OR malignant hypertension) OR severe hypertension |
| Search ((((((((((((("Africa"[MeSH] OR Africa*[tw] OR Algeria[tw] OR Angola[tw] OR Benin[tw] OR Botswana[tw] OR "Burkina Faso"[tw] OR Burundi[tw] OR Cameroon[tw] OR "Canary Islands"[tw] OR "Cape Verde"[tw] OR "Central African Republic"[tw] OR Chad[tw] OR Comoros[tw] OR Congo[tw] OR "Democratic Republic of Congo"[tw] OR Djibouti[tw] OR Egypt[tw] OR "Equatorial Guinea"[tw] OR Eritrea[tw] OR Ethiopia[tw] OR Gabon[tw] OR Gambia[tw] OR Ghana[tw] OR Guinea[tw] OR "Guinea Bissau"[tw] OR "Ivory Coast"[tw] OR "Cote d'Ivoire"[tw] OR Jamahiriya[tw] OR Jamahiryia[tw] OR Kenya[tw] OR Lesotho[tw] OR Liberia[tw] OR Libya[tw] OR Libia[tw] OR Madagascar[tw] OR Malawi[tw] OR Mali[tw] OR Mauritania[tw] OR Mauritius[tw] OR Mayote[tw] OR Morocco[tw] OR Mozambique[tw] OR Mocambique[tw] OR Namibia[tw] OR Niger[tw] OR Nigeria[tw] OR Principe[tw] OR Reunion[tw] OR Rwanda[tw] OR "Sao Tome"[tw] OR Senegal[tw] OR Seychelles[tw] OR "Sierra Leone"[tw] OR Somalia[tw] OR "South Africa"[tw] OR "St Helena"[tw] OR Sudan[tw] OR Swaziland[tw] OR Tanzania[tw] OR Togo[tw] OR Tunisia[tw] OR Uganda[tw] OR "Western Sahara"[tw] OR Zaire[tw] OR Zambia[tw] OR Zimbabwe[tw] OR "Central Africa"[tw] OR "Central African"[tw] OR "West Africa"[tw] OR "West African"[tw] OR "Western Africa"[tw] OR "Western African"[tw] OR "East Africa"[tw] OR "East African"[tw] OR "Eastern Africa"[tw] OR "Eastern African"[tw] OR "North Africa"[tw] OR "North African"[tw] OR "Northern Africa"[tw] OR "Northern African"[tw] OR "South African"[tw] OR "Southern Africa"[tw] OR "Southern African"[tw] OR "sub Saharan Africa"[tw] OR "sub Saharan African"[tw] OR "subSaharan Africa"[tw] OR "subSaharan African"[tw]))))))))))))))))))) |
| Search ((((((Prevalence) OR Incidence) OR Frequency)) AND (((((Chronic kidney disease) OR Chronic kidney insufficiency) OR Chronic renal failure) OR chronic renal insufficiency) OR CKD)) AND ((((Hypertension) OR High blood pressure) OR malignant hypertension) OR severe hypertension)) AND (((((((((((((("Africa"[MeSH] OR Africa*[tw] OR Algeria[tw] OR Angola[tw] OR Benin[tw] OR Botswana[tw] OR "Burkina Faso"[tw] OR Burundi[tw] OR Cameroon[tw] OR "Canary Islands"[tw] OR "Cape Verde"[tw] OR "Central African Republic"[tw] OR Chad[tw] OR Comoros[tw] OR Congo[tw] OR "Democratic Republic of Congo"[tw] OR Djibouti[tw] OR Egypt[tw] OR "Equatorial Guinea"[tw] OR Eritrea[tw] OR Ethiopia[tw] OR Gabon[tw] OR Gambia[tw] OR Ghana[tw] OR Guinea[tw] OR "Guinea Bissau"[tw] OR "Ivory Coast"[tw] OR "Cote d'Ivoire"[tw] OR Jamahiriya[tw] OR Jamahiryia[tw] OR Kenya[tw] OR Lesotho[tw] OR Liberia[tw] OR Libya[tw] OR Libia[tw] OR Madagascar[tw] OR Malawi[tw] OR Mali[tw] OR Mauritania[tw] OR Mauritius[tw] OR Mayote[tw] OR Morocco[tw] OR Mozambique[tw] OR Mocambique[tw] OR Namibia[tw] OR Niger[tw] OR Nigeria[tw] OR Principe[tw] OR Reunion[tw] OR Rwanda[tw] OR "Sao Tome"[tw] OR Senegal[tw] OR Seychelles[tw] OR "Sierra Leone"[tw] OR Somalia[tw] OR "South Africa"[tw] OR "St Helena"[tw] OR Sudan[tw] OR Swaziland[tw] OR Tanzania[tw] OR Togo[tw] OR Tunisia[tw] OR Uganda[tw] OR "Western Sahara"[tw] OR Zaire[tw] OR Zambia[tw] OR Zimbabwe[tw] OR "Central Africa"[tw] OR "Central African"[tw] OR "West Africa"[tw] OR "West African"[tw] OR "Western Africa"[tw] OR "Western African"[tw] OR "East Africa"[tw] OR "East African"[tw] OR "Eastern Africa"[tw] OR "Eastern African"[tw] OR "North Africa"[tw] OR "North African"[tw] OR "Northern Africa"[tw] OR "Northern African"[tw] OR "South African"[tw] OR "Southern Africa"[tw] OR "Southern African"[tw] OR "sub Saharan Africa"[tw] OR "sub Saharan African"[tw] OR "subSaharan Africa"[tw] OR "subSaharan African"[tw])))))))))))))))))))) |

**Supplementary Table S2:** Scoring criteria for quality of studies (Adapted from Stanifer et al. Lancet Glob Health 2014; 2 e174 – 181.

|  | **CRITERIA QUESTION** | **YES (1)** | **NO (0)** |
| --- | --- | --- | --- |
| 1 | Are the study participants representative of the population with hypertension in the country of study? |  |  |
| 2 | Did the study exclude individuals with other established risk factors for CKD? (These factors may include Hypertension, obesity, underweight and Diabetes) |  |  |
| 3 | Is the sample size adequate (1,000 will be regarded as adequate) |  |  |
| 4 | Were the study participants recruited at random? (Non-probability sampling methods will be considered inadequate) |  |  |
| 5 | Was the response rate at least 60% of the initial sample size? |  |  |
| 6 | Was the exclusion rate less than 10% of the total sample? |  |  |
| 7 | Was eGFR defined as being ≤ 60ml/min/1.73m^2^ using the MDRD/CKD-EPI or Cockroft-Gault formulae? |  |  |
| 8 | Were the sociodemographic characteristics of the study participants adequately characterized? |  |  |

Quality of study: High quality= 7 – 9; Medium quality, 4-6; Low quality – less than 4

**Supplementary Table S3:** Risk of bias assessment

| Author | Year | Country | Representative sample in country | Exclude other risk factors | Sample size adequate (1,000) | Random sampling | Response rate >60% | Exclusion 10% of sample size | eGFR <60 for CKD Definition | Socio-demographics well defined | Total | Quality |
| --- | --- | --- | --- | --- | --- | --- | --- | --- | --- | --- | --- | --- |
| Ayodele | 2007 | Nigeria | 0 | 1 | 0 | 0 | 1 | 1 | 1 | 1 | 5 | medium |
| Peer | 2008 | South Africa | 0 | 1 | 0 | 0 | 1 | 1 | 1 | 1 | 5 | medium |
| Osafo | 2011 | Ghana | 0 | 1 | 0 | 0 | 0 | 0 | 1 | 1 | 3 | low |
| Ajayi | 2014 | Nigeria | 0 | 1 | 0 | 0 | 1 | 1 | 1 | 1 | 5 | medium |
| Nelissen | 2014 | Nigeria | 1 | 1 | 0 | 1 | 0 | 0 | 1 | 1 | 5 | medium |
| Kabedi | 2014 | DRC | 0 | 1 | 0 | 0 | 1 | 1 | 1 | 1 | 5 | medium |
| Aryee | 2016 | Ghana | 0 | 1 | 0 | 0 | 1 | 1 | 1 | 1 | 5 | medium |
| Peck | 2016 | Tanzania | 1 | 1 | 0 | 1 | 0 | 0 | 1 | 1 | 5 | medium |
| Kalyesubula | 2017 | Uganda | 1 | 1 | 0 | 1 | 0 | 0 | 1 | 1 | 5 | medium |
| Adjei | 2018 | Ghana | 1 | 1 | 0 | 0 | 0 | 0 | 1 | 1 | 4 | medium |
| Ploth | 2018 | Tanzania | 1 | 1 | 0 | 1 | 0 | 0 | 1 | 1 | 5 | medium |
| Tannor | 2019 | Ghana | 0 | 1 | 1 | 0 | 0 | 0 | 1 | 1 | 4 | medium |

**Supplementary Figure 1:** Funnel plot to assessing publication bias (ES - Effect Size)


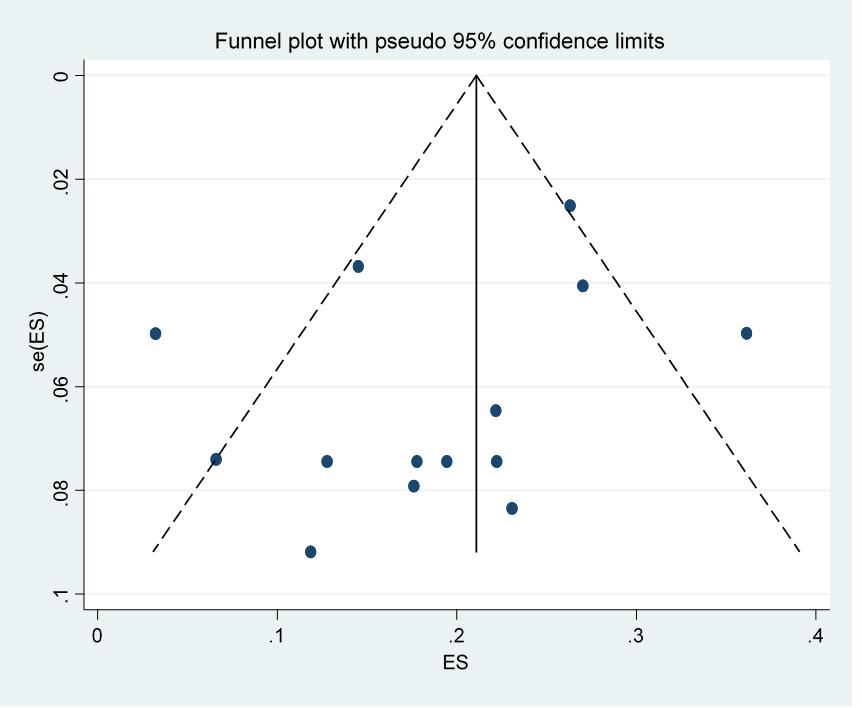


**Supplementary Figure 2**. Prevalence of CKD categorized by age groups

(ES - Effect Size; CI - Confidence Interval)


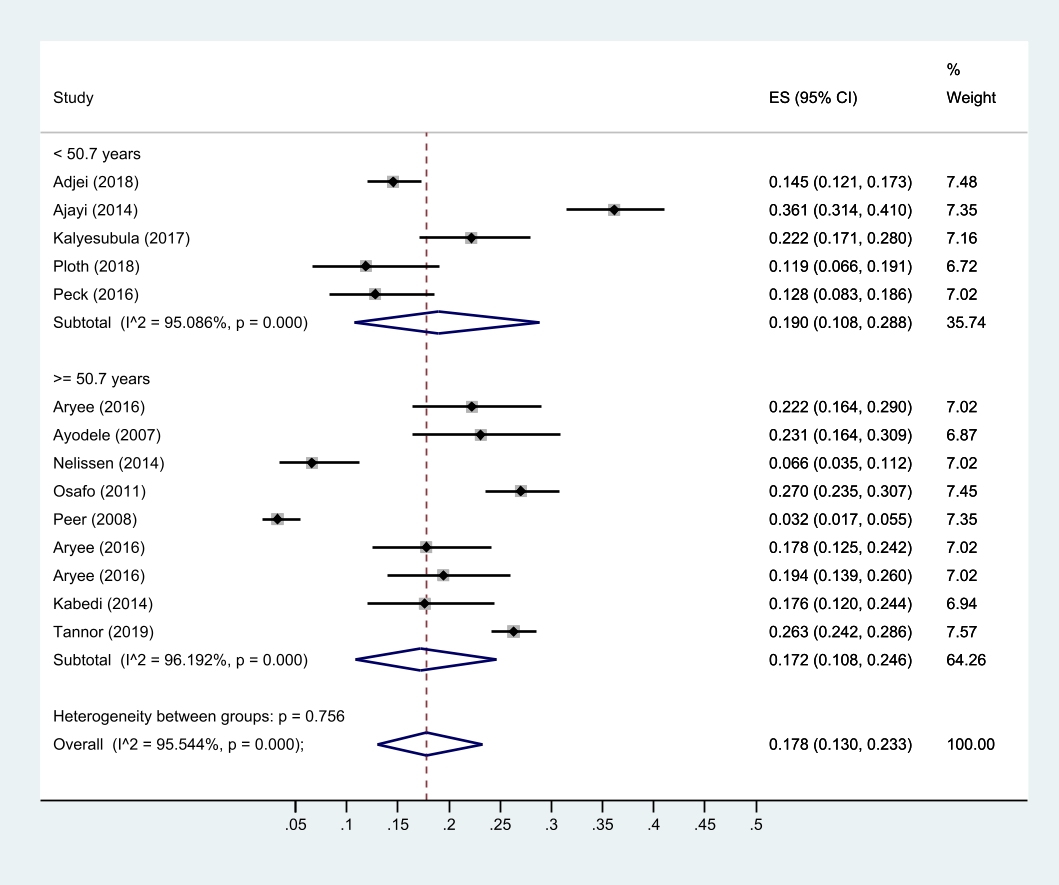


**Supplementary Figure 3: . Prevalence of CKD categorized by female gender preponderance (ES - Effect Size; CI - Confidence Interval)**

**
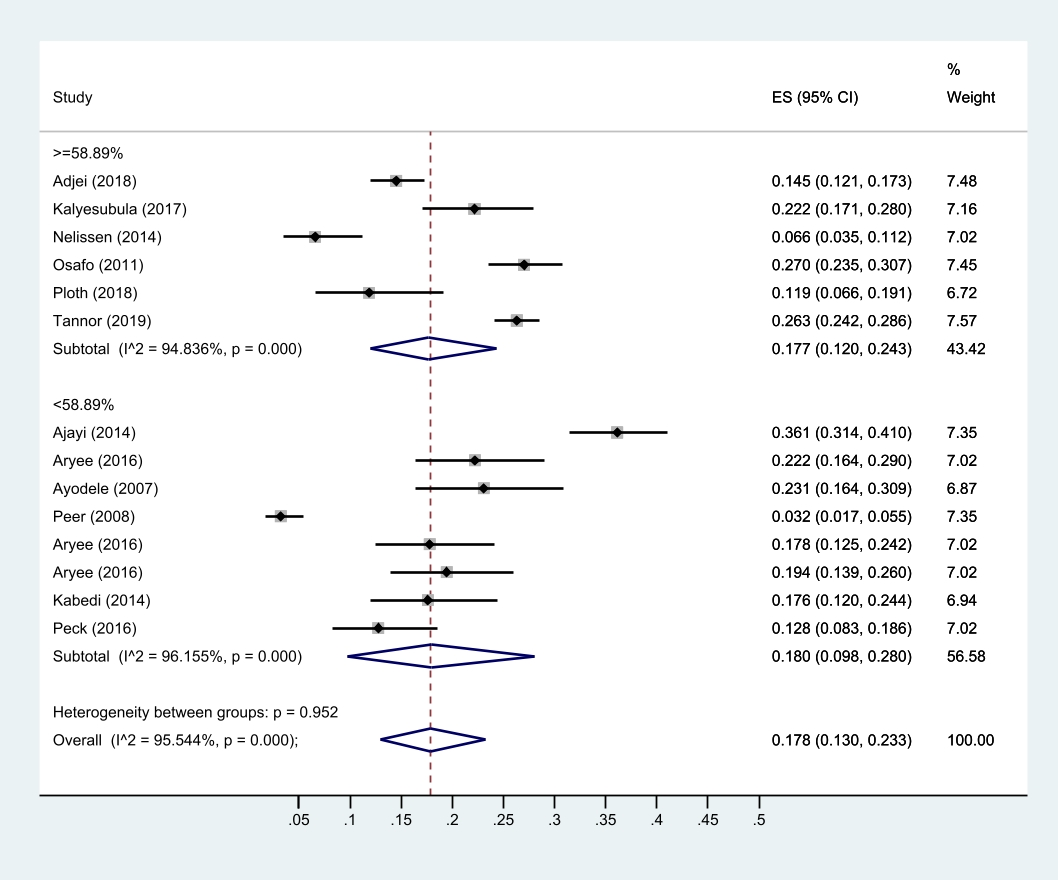
**
